# Considering patient-centred care and patient empowerment as essential to quality health care delivery in Ghana

**DOI:** 10.4102/hsag.v29i0.2608

**Published:** 2024-11-14

**Authors:** Ruby V. Kodom, Robert T. Netangaheni

**Affiliations:** 1 Department of Health Studies, College of Human Sciences, University of South Africa, Pretoria, South Africa; 2 Department of Health Services Management/DE, School of Continuing and Distance Education, University of Ghana, Accra, Ghana

**Keywords:** patient-centred care, patient empowerment, Universal Health Coverage, healthcare service delivery, health systems, quality service experience

## Abstract

**Background:**

Rising cases of non-communicable diseases (NCDs), poor health choices, mistrust of the health system, self-medication, resistance of diseases to medication and dissatisfaction with the service experience serve as red flags on the path to Universal Health Coverage (UHC).

**Aim:**

This study explored the importance of patient-centred care (PCC) and patient empowerment (PE) within the context of healthcare service delivery in Ghana.

**Setting:**

The study considered PCC and PE from the perspective of three public healthcare facilities within the Greater Accra region.

**Methods:**

The researcher adopted a qualitative exploratory research approach. The study employed purposive sampling for the selection of healthcare facilities and healthcare providers. Convenience sampling was applied to select patient participants. A total of 33 in-depth interviews and 4 focus group discussions (FGDs) were conducted across the three sampled facilities as part of the data collection process.

**Results:**

Healthcare providers and patients unanimously view quality as essential for effective healthcare delivery. The application of the capacitating role of PE and PCC is inexact and largely left to subjective interpretation.

**Conclusion:**

The Ministry of Health (MOH) has recognised that patient experiences, a key indicator of care quality, have often been suboptimal and is actively working to address these weaknesses.

**Contribution:**

This study supports the call for health systems to remain committed to efforts to achieve UHC, with a focus on PCC and PE.

## Introduction

The evolution of healthcare delivery in lower-middle-income settings such as Ghana is particularly complex because of the simultaneous burden of infectious diseases and the rising prevalence of non-communicable diseases (NCDs), both of which are competing for healthcare resources and attention (Kushitor & Boatemaa 2018). The rise in chronic cases has implications for health service providers and patients, who find themselves in a relationship of co-creation of care (Amu et al. 2021; Li et al. 2022). The provider–patient interaction in Ghana comprises patients who require care and healthcare providers who address that need by providing care. Clinical care has largely been viewed as a biomedical tool for health that focusses on curing diseases and not necessarily addressing patient needs. Studies on patient care experiences are few within the Ghanaian context (Sokoloff et al. 2020) and by extension that of Africa (Camara et al. 2020; Geldsetzer et al. 2018).

In a study conducted in Ghana, the author found that although patients reported positive experiences with the healthcare delivery received, they cited inequities in responsiveness by healthcare providers. Patient satisfaction affects treatment outcomes (Ofei-Dodoo 2019).

Quality in healthcare having been recognised as a pillar for attaining Universal Health Coverage (UHC) is difficult to quantify and measure. The Institute of Medicine’s (IOM) definition of quality enumerates six core pillars of quality and central to these pillars is patient-centred care (PCC) (Institute of Medicine, 2001). Without the patient, there would be no healthcare service delivery. Studies have observed that the experiences of patients within the sub-Saharan African region, where Ghana is located, do not reflect well on the objectives of the practice of health care. It is anticipated that clearly defining what quality healthcare means would contribute to the development of healthcare services and institutions that build trust with patients while creating a society of healthy and productive citizens (Geldsetzer et al. 2018). As concluded by Geldsetzer et al. (2018), UHC will be achieved if steps are taken to strengthen health systems in Africa with an emphasis on quality care. Africa’s health agenda invites action around creating awareness to engineer systemic changes which project PCC that espouses local values for interpersonal interaction.

The Ghana Health Service (GHS), in partnership with USAID, developed a strategic plan for quality assurance in 2007 (Ghana Health Service, 2007). This document provided a framework for care delivery that emphasized client-centeredness. Furthermore, the GHS has ‘people-centered’ as one of its core values and as an ideal state of health care quality in Ghana (MOH 2016). This study sought to contribute to the efforts at designing a home-grown version of PCC by first unearthing the prevailing conditions underpinning how the concepts of PCC and patient empowerment (PE) feature in healthcare service delivery (Agyepong et al. 2017).

Universal health coverage is measured by the proportion of a population that can access essential quality health services and the proportion of the population that spends a large amount of household income on health (Jensen, Kelly & Avendano 2022; WHO 2022). The underlying word quality underscores the assertion that access, wherever and whenever it is made available should be of quality.

The problem of inequitable UHC and access stems from a fundamental problem of poor-quality care. This is shown in developing settings by a lack of awareness and understanding of PCC and PE as healthcare quality concepts essential to practice (Ofei-Dodoo 2019). Patient-centred care is one of the key domains of quality in healthcare, but the extent to which PCC is being utilised remains largely unexplored (Bokhour et al. 2018). Although this concept is known globally and not alien to Ghanaian society, an extensive assessment of the literature on PCC shows that this is a relatively new area (Nkrumah & Abekah-Nkrumah 2019). The interest in PCC and PE stems from studies highlighting a superficial understanding of these concepts despite their potential to enhance healthcare quality (Ahenkorah & Nicola 2015). In lower-middle-income countries (LMICs) such as Ghana, the benefits of these concepts have yet to be fully realised. Challenges such as unequal access to healthcare and increasing legal disputes against care providers underscore the current discontent with service delivery and emphasise the need for improved provider–patient engagement (Camara et al. 2020; Moyer et al. 2014). Research indicates that PE and PCC not only improve health outcomes but also reduce healthcare costs (Radwin et al. 2019). Thus, prioritising PCC is crucial for achieving comprehensive and effective healthcare delivery.

As a developing nation with competing demands and socio-economic pressures, it becomes imperative for Ghana to explore functional ways of meeting the health needs of the populace by making the responsibility for the population’s general well-being, a shared one. Ghana aims to achieve Sustainable Development Goal (SDG) 3, which focusses on ensuring healthy lives and promoting well-being for all ages (Lozano et al. 2019). However, current statistics indicate that Ghana is off track to meeting the targets set under SDG 3 (Sachs et al. 2022). Quality healthcare delivery is crucial to addressing this shortfall, as past failures in achieving Millennium Development Goals were attributed to deficiencies in healthcare quality (Escribano-Ferrer et al. 2016). Improving PCC and PE could enhance healthcare outcomes and reduce costs, thereby supporting Ghana’s progress towards SDG 3.

### Study purpose

The study primarily sought to investigate the awareness and application of PCC and PE among healthcare service providers and patients in Ghana. The specific objectives were:

1. To assess the understanding of the concepts of PCC and PE.
2. To explore the perceptions of quality healthcare among healthcare service providers and patients.
3. To analyse how the implementation of PCC and PE influences the quality of healthcare delivery in Ghana.

## Research methods and design

A qualitative research approach was employed with an exploratory descriptive design. This methodology allowed data to be collected in a manner that improved the understanding of the phenomena of PE and PCC through the lens of context in terms of people and their locations. In this study, the context was essential to understand the views of study participants and the gaps inherent as far as awareness of PCC and PE were concerned.

Exploratory design, as described by Hunter et al. (2018), provides a framework for investigating subjects that have limited prior reporting. This methodology allows researchers to delve into areas where existing literature may be sparse or fragmented.

The descriptive research design is employed to observe and describe the phenomenon under study. This methodological approach is instrumental in ensuring that the observations are purely reflective of participants’ real comprehension.

### Setting

The study was conducted at three health facilities in Accra, Ghana. These public health facilities provide health care services to mainly low-income communities in the capital.

### Population

The study population consisted of healthcare providers (nurses, doctors and administrators) working in the three identified health facilities in Ghana. The population also included patients seeking care at these three facilities.

### Sample and sampling method

The three health facilities (i.e. the Frafraha Health Center, the Greater Accra Regional Hospital and the Korle-Bu Teaching Hospital) were purposively selected as they provide healthcare services under Ghana’s Ministry of Health (MOH). The facilities are also aligned to the three-tier structure of service delivery in Ghana. The criteria for inclusion were that the facility had to be a public facility and identified as a provider of primary, secondary or tertiary care. Healthcare provider participants were selected purposively based on their positions in the three identified facilities Data saturation informed the sample size of the study participants. The number of years of experience of the sampled healthcare provider participants ranged from entry-level at under 2 years to 35 years. A total of 28 clients and/or patients contributed to the study as patient participants.

### Data collection and tools

The process of data collection for this study lasted from October 2022 to July 2023. Two sets of interview guides were used to moderate the interactions with the two groups of study participants. Individual face-to-face interviews with healthcare provider participants were used to collect data. The interviews were supplemented with audio recordings and researcher notes to enhance the data collection process.

To obtain the patient views, four focus groups were carried out face-to-face with care recipients in groups of seven at the three sampled facilities. Two sets of discussions were held at the regional hospital and one each at the health centre and the tertiary care facility. The focus groups with patients were conducted to address the study objectives by obtaining the lived experiences of patients about their understanding and experience of PCC and PE, exploring their perceptions of quality healthcare, and analysing how these concepts influence the quality of healthcare delivery in Ghana. The sessions lasted for 2 h on average. The researcher facilitated the sessions with the support of a research assistant. The sessions were found to provide clients and/or patients who were displeased with aspects of their experience an outlet for expression in a safe setting. A discussion guide of prepared questions ensured that conversations stayed focussed. The participants of this discussion were clients and/or patients who were willing and able to participate without any form of coercion. The discussions were conducted in the English language and the Ghanaian language, Twi.

### Data analysis

Data analysis was done using Braun and Clark’s (2006) six steps of thematic analysis. The 33 one-on-one interviews and 4 focus group discussions (FGDs) were used to generate the codes which were categorised and from which themes emerged. Reflection was activated as the researcher interacted with the data by listening to the audio recordings and making notes. The linkages drawn from this process informed the codes and categories that were generated for the development of sub-themes and themes.

### Trustworthiness

To ensure the study’s robustness, Guba’s (1990) model of trustworthiness, as noted by Flick (Dang, Nguyen & Thao Tran 2024), was employed, focussing on credibility, dependability, transferability and confirmability (Nieswiadomy & Bailey 2018). Credibility was maintained by documenting standard research methods, although member checking was incomplete because of resource constraints.

Data were gathered in healthcare participants’ work environments and during their daily activities to ensure prolonged engagement. For patient participants, data were collected in healthcare facilities and in quiet rooms when available.

Flick (Dang et al. 2024) emphasises data triangulation for validating research. This study used interviews, research field notes, audio recordings and secondary sources including relevant journal articles, government reports and scholarly publications. Confirmability was achieved through triangulation and careful data handling, employing bracketing and use of quotations.

Dependability was ensured by using the researcher’s notes to reduce self-bias in conceptualisation and reporting (Mabbott et al. 2013). Transferability was addressed by detailing and contextualising information to allow readers to infer the applicability of findings to other contexts (Forero et al. 2018).

### Ethical considerations

For this study, the University of South Africa (UNISA) Research Ethics Committee (Higher Degrees Committee) of the College of Human Science reviewed the study proposal and granted clearance for the research (69969213_CREC_CHS_2021). The GHS Ethics Committee and the Korle Bu Teaching Hospital Scientific Research Committee gave authorisation for the study to be conducted. Permission was also sought and granted by the management of the facilities and clients and/or patients before data collection. Participants were assured that their anonymity and confidentiality would be maintained.

## Results

A total of 33 healthcare service providers with varying years of experience as nurses, doctors and administrators took part in this study. The aim was to engage different aspects of the service delivery channel using the lens of these study participants. Furthermore, 28 patient views were also obtained through four focus groups that were conducted with patients in groups of seven.

Participants were identified based firstly on the order in which they were interviewed and the facility to which they belonged at the time of the study. Thus, the first participant from the Korle Bu Teaching Hospital (KBTH) is recorded as ‘R1’ (Respondent number 1); ‘IDI’ (In-depth Interview); ‘KBTH’ (facility). Thus, R1, IDI, KBTH. Responses from FGDs were represented as Respondent number, FGD, and Facility name. Hence, R1 FGD, Frafraha Health Centre (FHC). In this way, responses were more easily categorised based on the linkages that were observed, Greater Accra Regional Hospital (GARH) was also used as one of the identifiers. Thematic analysis was conducted separately for healthcare service providers and patients to provide a clear and in-depth understanding of each group’s experiences and perspectives.

## Healthcare service providers themes

### Theme 1: Awareness of patient-centred care

Primary healthcare which is healthcare that is decentralised has become of paramount importance given the rise in the incidence of NCDs globally. This signifies that African countries such as Ghana are now having to manage not only communicable diseases but predominantly lifestyle-related conditions. This is why awareness on the part of healthcare service providers must be topical so that all patient interactions are maximised to produce better outcomes through patients who exercise their authority to make the right choices for their health. The awareness of healthcare provider participants on the concepts of PE and PCC was mixed. Generally, there was a fundamental appreciation of PE as patient education and PCC as care that is focussed on addressing the needs of the patient as an individual. There were a few instances where healthcare providers were not aware of both PE and PCC. In comparing the two concepts, it was also found that healthcare providers were more familiar with PCC than with PE.

#### Sub-theme 1.1: Understanding of patient-centred care

Patient-centred care is care that recognises the patient as a person. Of the 33 in-depth interviews conducted, almost all participants assented to having heard about the term, ‘patient-centred care’.

The responses from a cross-section of health workers are outlined as follows:

‘Patient-centred care has to do with the quality service rendered to clients when they come to the facility. So, the type of care you give, it should involve the patient and then it has to have the component of err the Ghana Health Service principles that we work with.’ (R2, IDI, FHC)

‘When we say patient, who is a patient? Somebody who is sick and needs help. What is the centred care? So, the care must be centred on the preferences and the needs of the patients.’ (R14, IDI, GARH)

‘Patient-centred care is about the individual… there should be a designated let me say a medical team that will have to take care of his health needs in totality. So, there is in the health team, there is a nurse, there is a doctor … even whenever he comes and there is a team member that is not even available, the other team [*members*] kind of have an idea of his [*c ondition*] and would be able to what, manage him as such.’ (R5, IDI, KBTH)

From the above-stated responses, it is apparent that generally, health workers in Ghana have some understanding of PCC as being care that ultimately considers the patient. Thus, the patient is priority to the provider–patient interaction.

#### Sub-theme 1.2: Introduction of patient-centred care as part of training

Study participants recalled with varying levels of certainty of having been taught about PCC as part of their educational preparation. This variability suggests that while PCC is included in the curriculum, its emphasis and the effectiveness of the training may vary significantly. Some quotes to support this are as follows:

‘Yes, we were taught in school. but it wasn’t for … not like a whole course something. Something little.’ (R3, IDI, FHC)

‘Yes, now you know, curriculum are being revised here and there but the common term that we hear, customer care. When you come to the health facilities that is what we use.’ (R14, IDI, GARH)

‘I think as part of my training … just getting to the end of the Degree programme.’ (R5, IDI, KBTH)

On the other hand, some participants did not express confidence about knowing what patient-centred care is:

‘[*N*]obody gave you quality control or quality assurance lectures … It was not part of the curriculum. But after school, you know you have to learn them, the hospital like puts in a lot to upgrade us.’ (R1, IDI, GARH)

‘Not that I remember.’ (R8, IDI, FHC)

There was at least one participant from each of the three selected hospitals who had not heard about PCC. This indicates a need for more consistent and comprehensive integration of PCC principles in healthcare education programmes to ensure that all healthcare providers have a good understanding and are adequately prepared to implement these concepts in practice.

### Theme 2: Capacitating role of patient empowerment as viewed by healthcare service providers

Health workers who participated in the study regarded PE as patient education. The responses sampled support this assertion as follows:

‘[*T*]he little time I have with them I make sure, they know what, they know what their condition is all about, yeah, proper health education. I mean, I give them proper health education.’ (R8, IDI, GARH)

‘I think it’s basically about educating them. Because I see a lot of patients even having influence on their own, their fellow patients having influence on their relatives … the only thing is that make sure the information you are giving them is correct … because if it’s correct, they spread it and spread it well.’ (R10, IDI, GARH)

‘the first point of contact is that we try to give education to the patient, and then after educating the patient on the procedures … we try to now give them another talk on any most current health issues or concern.’ (R5, IDI, KBTH)

The responses from participants on PE suggest that the understanding is inclined towards the construct overlap of PE as education.

While it is good to find that Ghanaian health workers are passionate about empowering patients by letting them know about their condition, it is even more important to have these patients placed in a position of power to make the right decisions that further good health in our communities.

#### Sub-theme 2.1: Importance of patient empowerment to healthcare service delivery

Patient empowerment is about allowing patients to have a say and exercise authority in decisions and choices made about their health. The benefits of an empowered patient are increased population health literacy, an awareness of the socio-cultural factors that impact on health and better adherence to medical strategies as a result of good patient–provider partnership. The patient has a lot of value to bring to the table where health-seeking is concerned. It is time to encourage patients to take up the responsibility that is so rightfully theirs. As the participants state:

‘[*T*]he other thing is letting our clients know the importance of seeking help so that they don’t stay for long in the house for it, for the disease or whatever is happening to them to get out of hand before they step out to seek care. So, if our clients know that if something is wrong with me, I am supposed to go and seek help. Then our work will be less difficult.’ (R6, IDI, FHC)

‘[*T*]hey will know that okay am not supposed to do a, b, c, and they will also follow it as well. So, you will see that even the hospital will not be congested because patients are now trying to be conscious of their health and their lifestyle.’ (R5, IDI, KBTH)

‘[*If*] I know how to take care of myself and even to also notice when something is going wrong, I don’t wait for it to deteriorate before I come to Ridge hospital and at that point becomes difficult to take care of or it will even cost more The instance of a client who is not enabled or empowered is cited in the following participant’s statement.’ (R5, IDI, GARH)

The healthcare provider participants across the three sampled facilities seem to agree that having empowered patients is beneficial to the work of nurses and doctors, and could produce more positive outcomes for patients. In transitioning from a paternalistic model of care to a patient-centred approach, healthcare workers often face challenges, particularly when patients are unwilling to engage actively in their care. This reluctance can be frustrating for healthcare providers who are trying to implement patient-centred practices. For instance, one health worker expressed their frustration with a patient who consistently refuses to adhere to prescribed treatment. The health worker shared:

‘There is one man in this school, anytime he comes here you have to talk a lot … He always comes like 171/110 [*BP*] and he’s like I’m becoming impotent. I cannot father a child. He’s been giving me flimsy, funny, funny excuses why he won’t take the medicine.’ (R3, IDI, FHC)

This example illustrates how patient resistance can complicate the shift towards a more collaborative and participatory model of care, highlighting the ongoing challenges in achieving PCC.

The benefits of having patients who understood their health needs also demonstrated the issue of PE from the perspectives of the patients. The patients in the FGDs described how being informed could protect them from likely harm. The extract that follows supports this:

‘I’ve finished my injections. She [*a nurse*] came and stood there, and she said I’m going to take another injection. I said ah! but my doctor said that I had finished with the injections. She said “No, no, no, you are going to”. I said no. Me; I know what I’m about. So, you can’t just come and do anything to me. so, she checked the book and said oh, sorry it’s not you.’ (R2, FGD, GARH)

No country can fully afford the cost of curative care which is why health promotion through PE and by extension PCC, can simply not be overlooked.

### Theme 3: Elements of quality in healthcare as comprehended by healthcare service providers

Quality of care or the lack thereof is identified as one of the reasons why Ghana failed to meet the health-related Millennium Development Goals (MDGs). Quality is vital as far as healthcare is concerned, and participants of the study in their one-on-one interviews assented to this as is captured in the responses presented as follows:

‘These 3 A’s, availability, accessibility, and affordability. People look at quality in that way.’ (R8, IDI, GARH)

‘Confidentiality, attentiveness [*active listening skills*], honour a customer’s time, preference and humility.’ (R1, IDI, FHC)

The responses that follow affirm that health workers appreciate the elements of quality outlined by the IOM as ‘safe, effective, patient-centred, timely, efficient and equitable’.

About timeliness, the study participants stated:

‘Waiting time: if you are not feeling well you don’t want to be sitting there the whole day waiting for help to come.’ (R13, IDI, GARH)

‘the environment also plays an optimum effect on patient recovery. So, it depends on the environment, then staff interaction with the patient and then the quality of the services that they kind of like access in the facility as well.’ (R5, IDI, KBTH)

These comments capture the need for key elements of quality to be present as understood by a provider participant highlighting, patient safety, cordial provider and/or patient engagement and efficiency in the delivery of care.

#### Sub-theme 3.1: Health worker perspective of service delivery interaction

Quality requires coherence between policy and practice. The disconnect between developed ideas about how service should be delivered and what is executed in practice, when removed, would go a long way to bring about improved, safe and quality healthcare. In health fundamentally, service should be viewed as synonymous with quality:

‘Quality care is about the patient or a client coming to the facility, at least getting what he or she wants at an affordable rate then at a time that will not keep the client waiting for long and where there’s space for the client to express him or herself.’ (R7, IDI, FHC)

Quality takes on different meanings depending on who is asked. That notwithstanding, some basic principles or elements are universally acceptable:

‘Quality in terms of availability of what I’m looking for, the service, is it available? So, people term quality, when I go to ABC Hospital, there is ART there, there is a family planning, there is a physician that when I have BP or diabetes so one time stop.’ (R14, IDI, GARH)

Participants therefore, talk about the right service, at the right time, for the right patient:

‘If we don’t give them quality care, they just keep coming back. Then we are not resolving the issues, so a lot of time is wasted, a lot of money is wasted.’ (R13, IDI, GARH)

Quality of care is a difficult concept to define. Perhaps this has contributed to the elusiveness of it being achieved universally. This is where Donabedian’s structure-process-outcome theory proved useful for attempting to understand the existing environment and structure, and the connected steps that result optimally in a healthier, satisfied patient. The reality remains that Ghana and other developing countries do not have the resources to fully cover and distribute health care services equitably. Ensuring that the right type of care is made available is an important way of addressing the failures of healthcare service delivery, and this is where quality comes into play.

## Patient themes

### Theme 1: Awareness of patient-centred care

From the client and/or patients’ perspective, the awareness level of the concept among patient stakeholders indicates that it is generally viewed as ‘customer care’ (R4 FGD KBTH):

‘I think they should work on, like, their care. I don’t know how to say it. With what they show, if it was their mom or sister or someone they knew, I don’t think they would talk the way they do, or you know, handle them the way they do. So, I’m sure they learnt all that in nursing school but putting it into practice is the hard part.’ (R4, FGD, KBTH)

Despite the efforts introduced to improve PCC in Ghana, the patient experience of care remains poor because of long waiting times, short consultation times, demand for unauthorised payments and a lack of respect. The study found that although the Ghanaian patient values respect and responsiveness to patient needs as primary indicators of PCC, they report not receiving this type of care as is captured in the extracts inserted here:

‘[*B*]ut I thought because they say patients are right or customers are right. But for him, he threw back. I haven’t seen a nurse having altercations with a patient before.’ (R4, FGD, KBTH)

#### Sub-theme 1.1 Patient knowledge of patient-centred care

Patient-centred care is loosely understood by patient as care that prioritises the patient but from the viewpoint of the patient as a client or customer. The following extract shows patients’ need to have a good relationship as customers:

‘there should be a customer relationship. There should be a better relationship between you [*healthcare givers*] and the client.’ (R3, FGD, GARH)

‘[*S*]o basically, patient-centred care is when you go to a health facility, what you have need of is what the healthcare providers should do for you … About you the patient, the client coming to the hospital.’ (R7, FGD, FHC)

It appears this association is the result of the institutional representation of PCC as ‘customer care’. In Ghana, patients value patient-centredness that focusses on respect and responsiveness to patient needs, preferences and values. There should be a concerted effort to ensure a patient-centred process for delivering healthcare in Ghana.

### Theme 2: Awareness of patient empowerment

Empowerment intrinsically deals with the creation of a patient who is able, willing and can exercise control. This means that the individuals are aware of their rights and responsibilities first to themselves, their families and their care providers.

During the FGDs with patients, a participant emphasises the role of PE as client education for good health outcomes:

‘[*O*]ne, the patient should be given information about his or her condition. Patients don’t like, she said ‘get drugs, go and take’. What am I taking it for? The side effects of the medication should be told to the patient such that maybe if you take this and you see this symptom, they are the side effect so then stop taking it and come back to the facility. Those people are not told. Else they are taking it and they are getting worse off instead of rather getting health, you are getting worse off.’ (R7, FGD, FHC)

The understanding of PE among patients is found to be skewed towards education and/or health literacy among client and/or patient stakeholders:

‘A lot of patients are ignorant about the patient’s rights and stuff. So, they should make that kind of information accessible to all especially, those who cannot read. They should try educate so that they can always know what to do.’ (R1, FGD, KBTH)

#### Sub-theme 2.1 Patient knowledge and/or experience of patient empowerment

Patient empowerment, although not widely understood, is also regarded as part of ‘customer care’ or as simply ‘listening to the patient’ (R4, FGD, KBTH). Gleaning from this premise, patient participants did not generally feel as empowered as patients because the ‘customer care’ experience was found to be lacking. For example, a focus group participant had this to say:

‘No. You are discharged, you are fine, the baby is fine, go home, bye-bye.’ (R6, FGD, GARH)

A study on responsiveness to healthcare interventions among women in Ghana found that scores for autonomy were low. This current study’s finding shows that this condition still remains. This is evident from the following comment where the participant expresses a sense of powerlessness:

‘[*B*]ut the thing is sometimes when you come, you don’t even know what is wrong with you. And the person too is not explaining to you. How would you even ask what this medication is going to do for you?.’ (RI, FGD, GARH)

Thus, PE as a process input appears not to be functioning well within this setting. Patient empowerment is not only the outcome of ‘an empowered patient’ but is also an important process in ensuring that positive health gains are sustainable.

### Theme 3: What quality means

Quality in healthcare is anchored on six domains as put forth by the IOM. The elements of person-centredness, timeliness, efficiency, effectiveness, safety and equity are what determine whether the healthcare services being provided are of good quality. For UHC to be achieved, it is imperative that LMICs such as Ghana, focus on incorporating these elements in the delivery of health services that meet the social, environmental (physical) and economic needs of the population. To articulate their views as patients, the following extracts are presented:

‘[*F*]or quality, one, I look out for the kind of facility you have in terms of lab. I look out for doctor-patient ratio. because I’ve been to, ok, a bigger hospital. You realise that you waste more time because the patient-doctor ratio or the midwife-patient ratio is so high.’ (R7, FGD, FHC)

Participant R7 went on to add that:

‘[*G*]ood communication from the midwives. when anything is wrong, you realise they don’t scare you, like they try and teach you for you to understand the dos and don’ts for you to get it right.’ (R7, FGD, FHC)

This shows that for patients, quality is defined by care that is responsive to their needs, embodies good communication and is respectful to their human dignity.

#### Subtheme 3.1: The mediating role of patient-centred care and patient empowerment on quality service delivery, patient perspectives

The IOM identified patient-centredness as a key dimension of healthcare where quality is concerned. The process component of Donabedian’s model of quality depicts how desired health outcomes are derived. Service delivery that takes account of the patient-centredness of care and by extension the resulting empowerment of individuals and their families contributes to continuous quality improvement. The authors are of the view that PE and PCC as processes produce long-term benefits which include appropriate lifestyle changes, enabled and informed patients, and sustained co-creation of responsive care:

‘[*T*]he communication between the patient and either nurse or the doctor. Some of the doctors make you the patient express what bothers you so that they explain to you. But some others, it seems what the doctor says is final. He doesn’t listen to the point of view of the patient.’ (R3, FGD, FHC)

‘When we go to the hospital, at least, they should ask us our concerns. They should ask what’s wrong or bothering us because I know myself and I’m able to tell what’s bothering me. Fine, you can go for check-up, you will be examined, the machine will examine you. But the machine is a machine. It can make errors… So, if you ask me then I can describe well. You can prescribe the right medicine for me.’ (R1, FGD, FHC)

‘[*H*]e[*doctor*] changed my medication, the dosage and didn’t tell me that this is the new dosage I’m supposed to be taking. And I went on with the old until I came for the next review. No, the medicine even got finished. When I went to the pharmacy to buy, then at the pharmacy they told me that this is the right dosage for me to be taking. By then I have already taken the old one, the old dosages for a very long time … assuming it was going to harm me in a way I will be dead, or I would have died by now.’ (R1, FGD, KBTH)

Patient-centred care creates empowered patients who are enabled to function as partners in the quest for better healthcare delivery. It appears that not enough attention is being given to the implementation of PCC and PE. It is important to note that patients have agency and can choose where to go to obtain desirable healthcare services.

### Theme 4: Communication as fundamental to implementing patient-centred care and patient empowerment

A cross-group analysis highlighted ‘communication’ as a common theme. Communication is an important aspect in the provider–patient interaction to enable PCC. From the patients’ viewpoint, communication with feedback from health workers made patients feel welcome and that they were listened to and their questions or concerns were addressed. A healthcare service provider had this to say:

‘[*Y*]ou see as for patients and their health care providers, the only way you empower them is good communication, very good communication and respect.’ (R12, IDI, GARH)

Both healthcare provider and patient groups emphasised communication as critical to quality care, but patients felt that communication was often lacking, indicating a gap between provider intentions and patient perceptions. The following extracts, one from a patient and the second from a provider, support this assertion:

‘[*T*]he communication between the patient and either nurse or the doctor. Some of the doctors make you the patient express what bothers you so that they explain to you. But some others, it seems what the doctor says is final. He doesn’t listen to the point of view of the patient.’ (R3, FGD, FHC)

‘I think communication means a lot and at times too if the patients are able to voice out, if there is a mutual communication between the staff and the patient, I think the patients are able to tell them what is actually wrong with them. Because at times we do counselling and when we are doing counselling, we can see that, maybe it’s not the condition that is killing the person but at times emotional stress and other stuff.’ (R2, IDI, KBTH)

It is when communication is built, that trust in the healthcare system is strengthened, for treatment care strategies to be successful.

## Discussion

To understand the role of PE and PCC among healthcare workers in Ghana, participants were asked about their understanding of these concepts and their influence on quality service delivery. The study explored healthcare providers’ knowledge of PE and PCC, and their perspectives on quality in healthcare. Participants’ comprehension of quality elements was examined to establish the connection between PE, PCC and healthcare quality. Although some participants recognised the link between these concepts and quality care, most adhered strictly to treatment guidelines, often sidelining holistic patient care. This observation aligns with findings from other studies. For example, a study that sought to develop a practical framework to guide the implementation of PCC (Santana et al. 2018) revealed that while healthcare providers acknowledged the importance of PCC, its implementation often lagged because of a predominant focus on clinical guidelines. Similarly, in studying interventions that promote PCC during consultations, Dwamena (2013) uncovered that despite understanding PCC principles, healthcare providers often prioritised biomedical aspects over holistic approaches. These findings suggest that while the theoretical importance of PE and PCC is acknowledged, their practical application remains challenging, necessitating further integration into daily clinical practice.

Zooming in on SDG3 on Good Health and Well-being, Ghana’s performance in national rankings (Sachs et al. 2022) underscores the need for renewed efforts to enhance the healthcare system and overall service delivery (Geldsetzer et al. 2018). The study uncovered mixed views on the concepts of PCC and PE, further complicated by structural challenges and resource constraints that hindered the delivery of PCC (Bokhour et al. 2018).

The findings from this study highlight the need for renewed commitment to UHC with a focus on PCC. This need is evidenced by the mixed understanding of PCC and PE among study participants and their roles in ensuring quality service delivery. Notably, many patients were unfamiliar with these concepts (Edgman-Levitan & Schoenbaum 2021). However, by clarifying the goals of care, this study that compared terms associated with PCC (Håkansson Eklund et al. 2019) found that with effective communication, training and structural interventions, patient involvement in care and quality outcomes could be improved. Healthcare providers in the study demonstrated some knowledge of PCC, as evidenced by the presence of pictorial displays promoting patient-centredness in sampled facilities (Nkrumah & Abekah-Nkrumah 2019). This emphasis on ‘customer care’ reflects the MOH’s efforts to improve patient experiences after recognising that the quality of care, as measured by patient experiences, has generally been found lacking (MOH 2021). Despite these efforts, some researchers (Afulani et al. 2020; Fix et al. 2018; Naik & Catic 2021) observe that there is insufficient scientific evidence to confirm that a community of PCC truly exists. More evidence is needed to demonstrate how healthcare systems operate, the processes that emphasise PCC and PE, and the outcomes supporting the widely held belief in the role these concepts play in improving population health outcomes. For instance, Grover et al. (2022) and Leidner et al. (2021) revealed a significant gap between what healthcare practitioners know and what they practice. This was evident in instances where, despite the presence of posters promoting PCC on hospital walls, both healthcare providers and patients largely overlooked them.

Regarding PE, the study found that understanding of the concept was often vague, with many equating it to patient education. In their studies on the concept of PE, Fumagalli et al. (2015) emphasise the need for clarity of definition so that patients can take up the agency that is being advocated. Furthermore, Acuña Mora et al. (2022) note that the difficulty in understanding PE arises from the varying connotations provided by different literature, leading to its interchangeable use with related concepts concerning patient action. The study’s findings align with the long-held notion of PE as a tool for health promotion, which the World Health Organization identifies as instrumental for global health (Acuña Mora et al. 2022). However, relegating PE to the background as irrelevant to biomedical care is problematic. While educating patients aims to create empowered individuals, it is not an automatic pathway to empowerment (Crondahl and Eklund Karlsson 2016). Thus, while it is encouraging to see that Ghanaian health workers are passionate about informing patients about their conditions, it is even more crucial to empower these patients to make informed decisions that promote their health (Halvorsen et al. 2020).

Quality is integral to UHC, as providing substandard services undermines the purpose of healthcare interventions, leading to additional costs and hardships for poor communities (WHO 2020). The IOM emphasised that quality should be the defining factor for 21st-century healthcare, with all healthcare organisations ensuring that care is safe, efficient, timely, patient-centred, effective and equitable (Institute of Medicine, 2001). Patient-centredness, central to these aims, cannot be separated from interventions aimed at providing access to quality healthcare services. Ghana’s Healthcare Quality Strategy Report for 2017–2021 defines quality by highlighting these aims and adds the dimension of ‘empowerment’ (MOH 2016). The report also links quality to achieving UHC, with Ghana’s vision of quality characterised by leadership, clear policies, evidence-based standards, motivated staff and client feedback for continuous improvement.

### Limitations

The study is unique in exploring quality, using PCC and PE as a lens for engaging health service providers, administrators and patients so that a balanced view of the lived experiences was obtained. The study, however, did not interrogate all the elements of quality in healthcare and cannot be deemed exhaustive on the subject of quality in healthcare. The qualitative nature of the study implies that although in-depth in profundity, the findings cannot be generalised without conducting further studies that cover a wider geographic location.

## Conclusion

It was reassuring to discover from this study that unanimously among patients and health care providers, quality was regarded as fundamental to the healthcare delivery apparatus. Quality care as concisely described by one health care provider participant is care that is accessible, available and affordable whenever it is needed. Resources be they financial or human are never enough. Employing quality in all aspects of healthcare delivery serves as a vital bridge between the population’s ill health and positive health outcomes. The cost of poor quality in health is too high a price for any person to bear. Thus, making available services that address the needs of the people is a mark of quality. Participants from either side of the aisle whether they be patients or health care providers hold the view that providing the right care, to the right patient, when it is needed, with the right skills, attitudes and medicines at a cost that is affordable by all is what constitutes quality healthcare. It is quite clear that providing access to quality healthcare does not entail simply putting up structures and equipping them with machines or medicines, but providing people with the right patient-centred services that lead to PE. Thus, there is a need for health systems to integrate and leverage PCC and PE beyond the cosmetic application of these concepts as it stands presently.
